# Supplementary material for: The gut microbiome-prostate cancer crosstalk is modulated by dietary polyunsaturated long-chain fatty acids
Source: Nat Commun. 2024 Apr 23;15:3431. doi: 10.1038/s41467-024-45332-w (PMC11039720; doi:10.1038/s41467-024-45332-w)
Supplement: Supplementary file 1 — Supplementary information [file 41467_2024_45332_MOESM1_ESM.pdf]

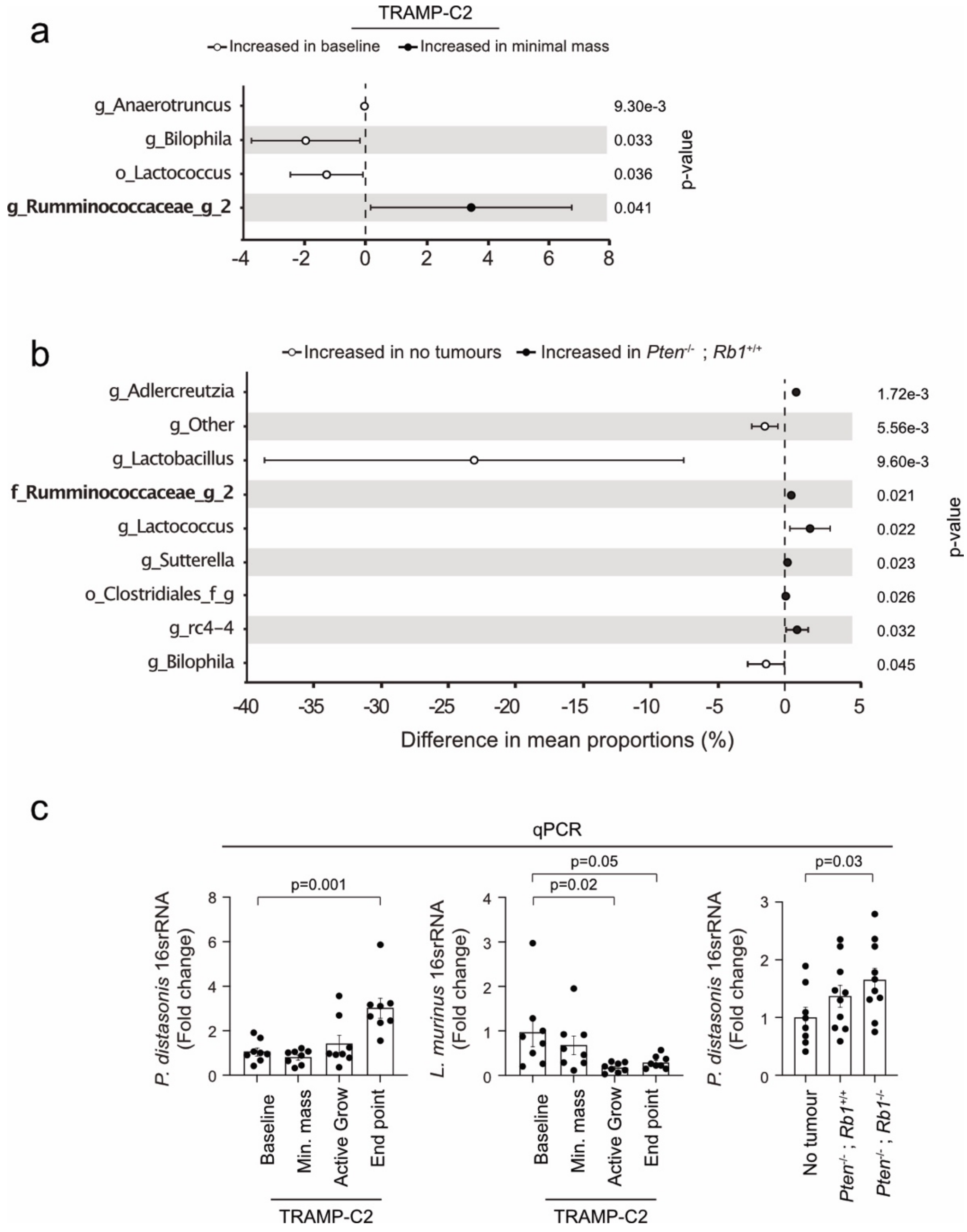

Supplementary Fig. 1. Differentially abundant bacteria at the genus level. **A** Fecal microbiota from mice with TRAMP-C2 tumours at the minimal mass growth stage were compared to the baseline before tumour cell injection (n=8 per group) using STAMP. **B** Fecal microbiota corresponding to mice with *Pten*$^{-/-}$; *Rb1*$^{+/+}$ tumours compared to tumour-free mice (n=8 per group) using STAMP. **C** Relative levels (fold change) of total 16S rRNA sequences corresponding to Parabacteroides distasonis (left, n=8 per group) and Lactobacillus murinus (middle, n=8 per group) from fecal samples of mice with TRAMP-C2 tumour harvested at different stages of prostate cancer development. Relative levels (fold change) of total 16S rRNA sequences corresponding to Parabacteriodetes distasonis (right) from fecal samples of mice without tumour, with *Pten*$^{-/-}$; *Rb1*$^{+/+}$ or *Pten*$^{-/-}$; *Rb1*$^{-/-}$, tumours (no tumour, n=8 and *Pten*$^{-/-}$; *Rb1*$^{+/+}$ or *Pten*$^{-/-}$; *Rb1*$^{-/-}$, n=10). Graphs in A) and B) are mean ± SD. Graphs in C) are mean ± SEM, p-values in A) and B) are from two-sided Welch's T test and in C) p-values are from two-sided Student's T test.

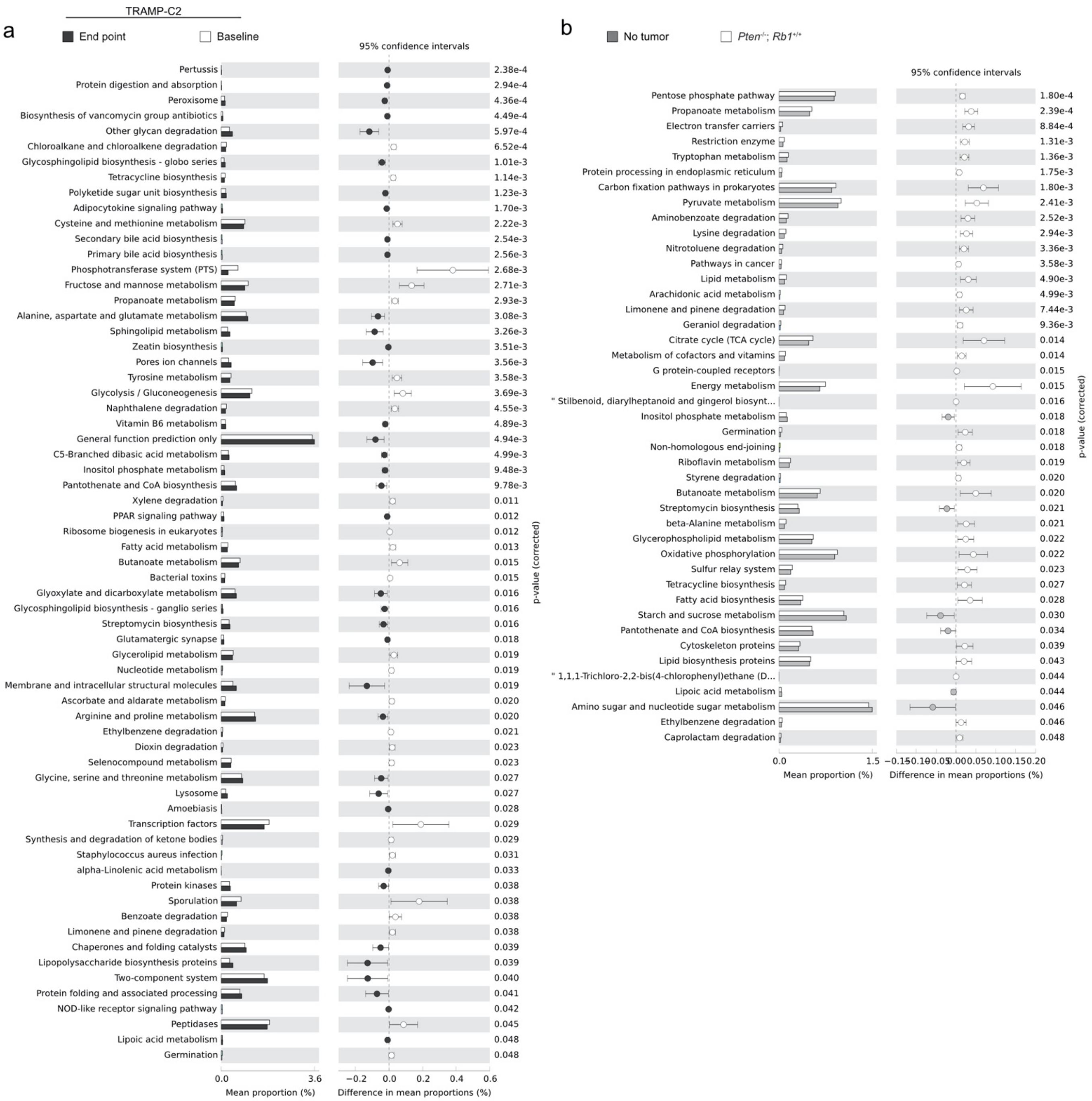

Supplementary Fig. 2 part 1. STAMP analysis of predicted functions. A Fecal microbiota comparison of mice with TRAMP-C2 tumours at the End point stage and baseline samples corresponding to main Fig. 2B. **B** Fecal microbiota comparison of mice with *Pten*$^{-/-}$;*Rb1*$^{+/+}$ tumours and no tumour samples corresponding to main Fig. 2E. Graphs are mean ± SD, =8 per group and p-values are from two-sided Welch's T test.

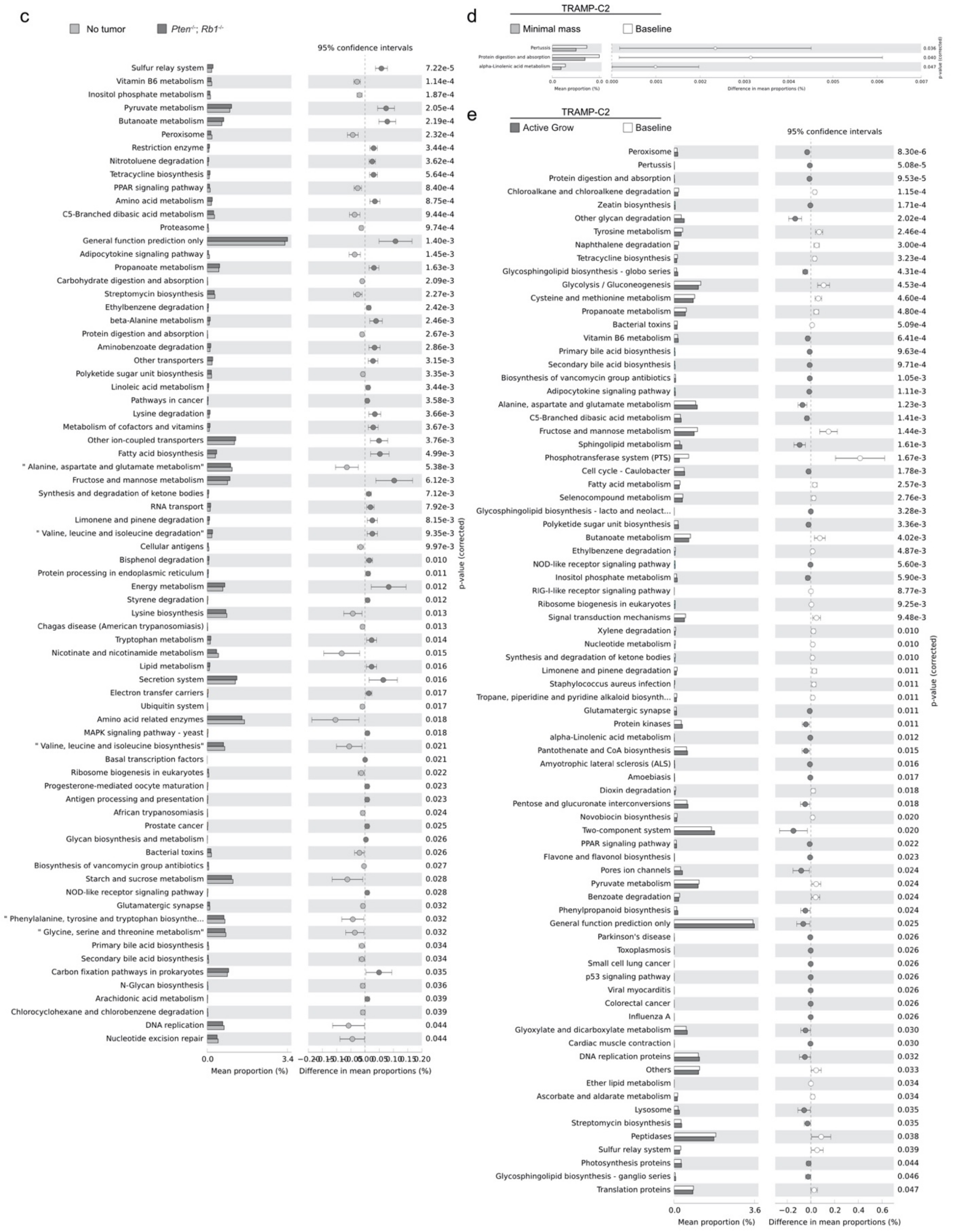

Supplementary Fig. 2 part 2. STAMP analysis of predicted functions. C Fecal microbiota comparison of mice with *Pten*$^{-/-}$;*Rb1*$^{-/-}$ tumours and no tumour samples corresponding to main Fig. 2E. **D** Fecal microbiota comparison of mice with TRAMP-C2 tumours at the Minimal mass stage and baseline corresponding to main Fig. 2B. **E** Fecal microbiota comparison of mice with TRAMP-C2 tumours at the Exponential growth stage and baseline samples corresponding to main Fig. 2B. Graphs are mean ± SD, =8 per group and p-values are from two-sided Welch's T test.

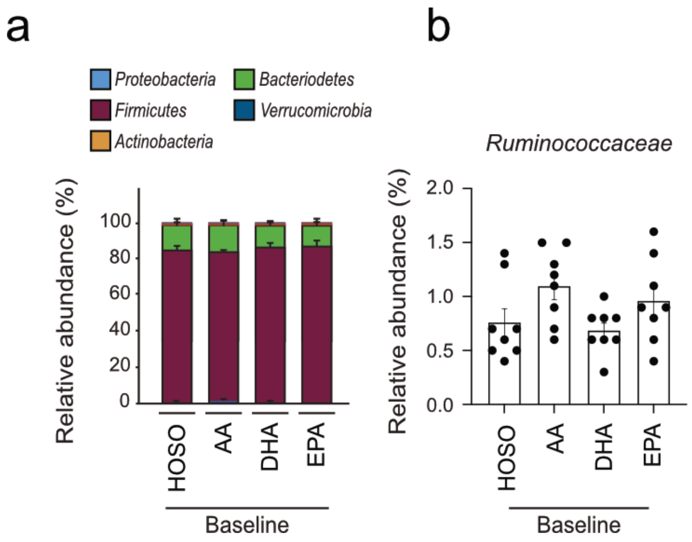

Supplementary Fig. 3. Baseline gut microbiota in a mouse model of prostate cancer development. A Relative abundance of baseline gut microbiota from mice before supplementation with high oleic sunflower oil (HOSO), monoglyceride arachidonic acid (MAG-AA), MAG-docosahexaenoic acid (DHA) or MAG-eicosapentaenoic acid (EPA) long-chain polyunsaturated fatty acids (PUFA) by oral gavage (n=8/group) at the phylum level. **B** Relative abundance of the 16S rRNA sequences corresponding to the Ruminococcaceae family (n=8/group). Data corresponds to the baseline conditions of Figure 4D. Graphs are mean ± SEM.

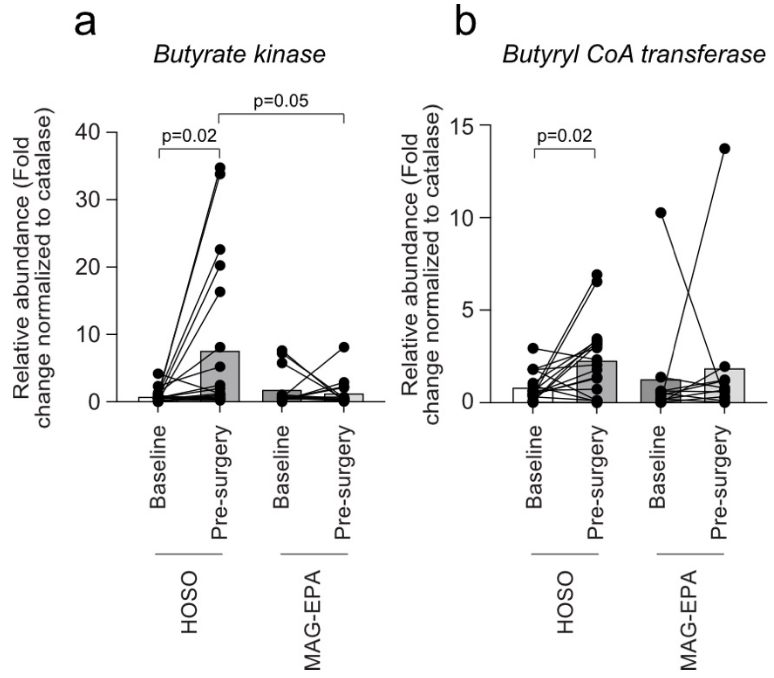

Supplementary Fig. 4. Short-chain fatty acid-related enzymes are modulated by a dietary intervention in prostate cancer patients. A randomized clinical trial (NCT02333435) was performed at our clinical facility testing the effect of MAG-EPA PUFA on prostate cancer patients before radical prostatectomy. A subset of 41 patients donated fecal samples for research. Using DNA extracted from fecal samples, we used qPCR to compare the profiles corresponding to men before and after 4-10 weeks of MAG-EPA (n=21) supplementation or HOSO placebo (n=20). The relative abundance of sequences corresponding to rate-limiting enzymes implicated in the biosynthesis of butyric acid (A and B) were measured by qPCR and normalized with bacterial catalase DNA levels. **A** The levels of Butyrate kinase and **B** Butyryl CoA transferase were compared patients receiving MAG-EPA or HOSO 7.2 ± 0.43 weeks prior to radical prostatectomy (Pre-surgery) and their levels at baseline. Graphs are mean ± SEM, two-sided Welch's T test.

Supplementary table 1. Clinical and pathological characteristics of patients pre-radical prostatectomy (corresponding to Figure 1 A-D).

**Tumor volume**<br>**Characteristic**	**Low [1]**<br>**(n=19)**	**Medium [2]**<br>**(n=20)**	**High [3]**<br>**(n=23)**	**p-value**
Age at radical prostatectomy (y.o)				0.75 [8]
Mean (SD)	66.8 (6.9)	65.3 (5.4)	66.5 (7.6)	
Body mass index (kg/m^2)				0.54 [8]
Mean (SD)	27.6 (4.6)	27.0 (3.3)	28.3 (3.8)	
PSA at prostate biopsy (ng/mL)				0.06 [8]
Mean (SD)	7.4 (9.3)	5.8 (3.2) [4]	16.0 (11.2)	
Min, max	1.1, 44.5	3.3, 9.8	4.5, 39.0	
Median (Q1, Q3)	5.7 (4.1, 6.5)	5.0 (4.4, 7.1)	12.4 (6.8, 21.0)	
PSA at radical prostatectomy (ng/mL)				0.16 [8]
Mean (SD)	6.6 (8.7)	6.1 (3.8) [5]	13.0 (9.4) [6]	
Min, max	0.9, 41.4	1.4, 16.0	3.7, 32.9	
Median (Q1, Q3)	5.0 (3.6, 6.1)	5.0 (3.9, 7.3)	10.2 (6.4, 18.1)	
ISUP grade group at biopsy, n (%)				0.42 [9]
1 (Gleason 6)	0 (0.0)	(0.0)	1 (4.3)	
2 (Gleason 3+4)	13 (68.4)	12 (60.0)	8 (34.8)	
3 (Gleason 4+3)	3 (15.8)	4 (20.0)	7 (30.4)	
4 (Gleason 8)	2 (10.5)	3 (15.0)	4 (17.4)	
5 (Gleason 9+)	1 (5.3)	1 (5.0)	3 (13.1)	
ISUP grade group at prostatectomy, n (%)				0.26 [10]
2 (Gleason 3+4)	13 (68.4)	13 (65.0)	10 (43.5)	
3 (Gleason 4+3)	3 (15.8)	5 (25.0)	5 (21.8)	
4 (Gleason 8)	1 (5.3)	1 (5.0)	1 (4.3)	
5 (Gleason 9+)	2 (10.5)	1 (5.0)	7 (30.4)	
Clinical T stage at biopsy, n (%)				**0.01** [11]
T2a or less	18 (94.7)	18 (90.0)	14 (90.0)	
T2b or T2c	0 (0.0)	2 (10.0)	7 (10.0)	
T3 or more	1 (5.3)	0 (0.0)	2 (0.0)	
Pathological T stage, n (%)				**<0.001** [12]
T2a or less	2 (10.5)	4 (20.0)	7 (30.4)	
T2b or T2c	15 (79.0)	9 (45.0)	3 (13.1)	
T3 or more	2 (10.5)	7 (35.0)	13 (56.5)	
Pathological N stage, n (%)				**0.01** [13]
N0	19 (100.0)	20 (100.0)	18 (78.3)	
N1	0 (0.0)	0 (0.0)	4 (17.4)	
N2	0 (0.0)	0 (0.0)	1 (4.3)	
Pathological M stage, n (%)		[7]		
M0	10 (52.6)	12 (60.0)	15 (65.2)	------
M1	0 (0.0)	0 (0.0)	0 (0.0)	
Mx	9 (47.4)	5 (25.0)	8 (34.8)	
Estimated tumor mass at prostatectomy (g)				
Mean (SD)	3.2 (1.4)	7.4 (1.4)	18.7 (12.7)	**<0.001** [8]

[1] Estimated tumor mass <5.5g calculated as follow: prostate weight (g) * % of tumor at surgery. [2] Estimated tumor mass ≥5.5g <10g. [3] Estimated tumor mass ≥10g. [4] One missing value. [5] 5 patients did not have a PSA measure at RP. [6] 6 patients did not have a PSA measure at RP. [7] 3 missing values. [8] Based on two-way ANOVA test. [9] Based on Exact Pearson Chi-Square Test, combining ISUP grade group 2 or less, as well as grade group 4 or more. [10] Based on Exact Pearson Chi-Square Test, combining ISUP grade group 4 or more. [11] Based on Exact Pearson Chi-Square Test, combining clinical stage T2b or more. [12] Based on Exact Pearson Chi-Square Test. [13] Based on Exact Pearson Chi-Square Test, combining clinical N stage N1/N2. Q: Quartile, PSA: Prostate-specific antigen, MAG-EPA: monoacylglyceride-conjugated eicosapentaenoic acid; ISUP: International society of urological pathology.

Supplementary table 2. Clinical and pathological characteristics of patients post-radical prostatectomy (corresponding to Figure 1 E-H).

BCR status<br>Characteristic	None [1]<br>(n=20)	Low PSA [2]<br>(n=9)	High PSA [3]<br>(n=18)	p-value
Age at RP (y.o)				0.10 [7]
Mean (SD)	67.0 (5.6)	63.9 (6.2)	62.6 (7.2)	
Body mass index at RP (kg/m^2)				0.53 [7]
Mean (SD)	27.9 (4.5)	26.5 (3.1)	26.9 (2.7)	
PSA at prostate biopsy (ng/mL)				0.54 [7]
Mean (SD)	15.2 (9.1) [4]	8.0 (6.7)	14.1 (24.0) [5]	
Min, max	4.7, 39.0	3.0, 22.0	3.7, 105.9	
Median (Q1, Q3)	8.4 (7.1, 16.4)	5.2 (4.3, 8.6)	5.2 (4.3, 8.6)	
PSA at RP (ng/mL)	NA	NA	NA	NA
ISUP grade group at biopsy, n (%)				0.32 [8]
1 (Gleason 6)	1 (5.0)	(0.0)	2 (11.1)	
2 (Gleason 3+4)	12 (60.0)	6 (66.7)	3 (16.7) [6]	
3 (Gleason 4+3)	3 (15.0)	2 (22.2)	6 (33.3) [6]	
4 (Gleason 8)	2 (10.0)	1 (11.1)	1 (5.6)	
5 (Gleason 9+)	2 (10.0)	0 (0.0)	3 (16.7)	
ISUP grade group at RP, n (%)				0.09 [8]
1 (Gleason 6)	0 (0.0)	0 (0.0)	1 (5.6)	
2 (Gleason 3+4)	15 (75.0)	6 (66.7)	5 (27.8)	
3 (Gleason 4+3)	2 (10.0)	2 (22.2)	4 (22.2)	
4 (Gleason 8)	1 (5.0)	0 (0.0)	4 (22.2)	
5 (Gleason 9+)	2 (10.0)	1 (11.1)	4 (22.2)	
Clinical T stage, n (%)			[5]	0.46 [9]
T2a or less	14 (70.0)	8 (88.9)	14 (77.8)	
T2b or T2c	5 (25.0)	1 (11.1)	2 (11.1)	
T3 or more	1 (5.0)	0 (0.0)	1 (5.6)	
Pathological T stage, n (%)				**0.01** [10]
T2a or less	9 (45.0)	1 (11.1)	1 (5.6)	
T2b or T2c	3 (15.0)	5 (55.6)	5 (27.8)	
T3 or more	8 (40.0)	3 (33.3)	12 (66.7)	
Pathological N stage, n (%)				0.06 [11]
N0	19 (95.0)	8(88.9)	12 (66.7)	
N1	1 (5.0)	1 (11.1)	4 (22.2)	
N2	0 (0.0)	0 (0.0)	2 (11.1)	
Pathological M stage, n(%)				
M0	10 (40.0)	4 (44.4)	16 (88.9)	------
M1	0 (0.0)	0 (0.0)	0 (0.0)	
Mx	10 (45.0)	5 (55.6)	2 (11.1)	
Estimated tumor mass at RP [4] (g)				0.99 [7]
Mean (SD)	12.9 (13.2)	13.2 (9.2)	17.5 (21.4) [5]	
Min, Max	4.5, 63.7	1.6, 32.0	0.9, 88.0	
Median (Q1, Q3)	8.6 (6.3, 13.7)	14.0 (6.8, 17.6)	9.9 (4.8, 17.2)	

[1] Patients with no BCR following surgery, [2] Patients who experienced ultrasensible BCR following a curative intent RP (PSA level <0.5ng/mL), [3] Patients who experienced a clinical BCR following a curative intent RP (PSA level >0.5ng/mL). [4] Estimated tumor mass calculated as follow: prostate weight (g) * % of tumor at surgery. [5] One missing value. [6] 3 patients had a Gleason 7 at BP, with no specification of primary and secondary pattern. [7] Based on two-way ANOVA test. [8] Based on Exact Pearson Chi-Square Test, combining ISUP grade group 2 or less, as well as grade group 4 or more. [9] Based on Exact Pearson Chi-Square Test, combining clinical stage T2b or more. [10] Based on Exact Pearson Chi-Square Test. [11] Based on Exact Pearson Chi-Square Test, combining clinical N stage N1/N2. Q: Quartile, PSA: Prostate-specific antigen, MAG-EPA: monoacylglyceride-conjugated eicosapentaenoic acid, ISUP: International society of urological pathology, RP: Radical prostatectomy, BCR: Biochemical recurrence.

Supplementary table 3. Clinical characteristics of donor patients for fecal microbiota transfers (Corresponding to Figure 3).

Characteristic	Donor 1	Donor 2	Donor 3	Donor 4	Donor 5	Donor 6
Age (y.o)	71.2	65.4	63.5	72.4	65.8	68.0
Body mass index (kg/m^2)	26.1	25.0	32.3	28.1	27.3	27.5
PSA (ng/mL)	44.48	5.91	4.82	20.27	6.69	11.63
ISUP grade group [1]	4	2	3	5	3	4
Cancer stage	T1c	T1c	T1c	T3	T1c	T2b
Estimated tumor mass at RP [2] (g)	1.2	3.4	9.4	10.0	10.3	21.0

[1] International society of urological pathology grade group: 2 = Gleason 7 (3+4), 3 = Gleason 7 (4+3), 4= Gleason 8, 5= Gleason 9+.
[2] Estimated tumor mass calculated as follow: prostate weight (g) * % of tumor at surgery. RP: radical prostatectomy, PSA: Prostate-specific antigen.

Supplementary table 4. Baseline characteristics of patients enrolled in the clinical trial (corresponding to Figure 5).

Characteristic	Placebo (n=20)	MAG-EPA (n=21)	p-value
Age (y.o)			0.97 [1]
Mean (SD)	65.8 (7.0)	65.9 (7.0)	
Body mass index (kg/m^2)			0.70 [1]
Mean (SD)	27.6 (4.5)	27.1 (2.9)	
PSA (ng/mL)			0.08 [1]
Mean (SD)	6.3 (4.4)	10.8 (10.4)	
Min, max	1.1, 23.0	2.3, 44.5	
Median (Q1, Q3)	5.7 (4.2, 6.6)	7.7 (4.7, 6.7)	
ISUP grade group, n (%)			0.47 [2]
2 (Gleason 3+4)	12 (60.0)	9 (42.9)	
3 (Gleason 4+3)	5 (25.0)	6 (28.6)	
4 (Gleason 8)	3 (15.0)	4 (19.0)	
5 (Gleason 9+)	0 (0.0)	2 (9.5)	
Cancer stage, n (%)			0.17 [3]
T2a or less	19 (95.0)	17 (81.0)	
T2b or T2c	1 (5.0)	2 (9.5)	
T3 or more	0 (0.0)	2 (9.5)	

[1] Based on two-sided Student's t-test. [2] Based on an Exact Pearson Chi-Square Test, combining ISUP grade group 4 or more. [3] Based on an Exact Pearson Chi-Square Test, combining cancer stage T2b or more. SD: Standard deviation, Q: Quartile, NCCN: National Comprehensive Cancer Network, PSA: Prostate-specific antigen, MAG-EPA: monoacylglyceride-conjugated eicosapentaenoic acid; ISUP: International society of urological pathology.

Supplementary table 5. Characteristics at prostatectomy of patients enrolled in the clinical trial (corresponding to Figure 5).

Characteristic	Placebo (n=20)	MAG-EPA (n=21)	p-value
PSA (ng/mL)			0.08 [2]
Mean (SD)	5.6 (4.2) [1]	9.9 (10.0)	
Min, max	0.9, 20.6	2.0, 41.4	
Median (Q1, Q3)	4.9 (3.8, 6.2)	6.34 (3.8, 6.1)	
ISUP Grade group, n (%)			0.84 [3]
2 (Gleason 3+4)	11 (55.0)	10 (47.6)	
3 (Gleason 4+3)	5 (25.0)	7 (33.3)	
4 (Gleason 8)	1 (5.0)	1 (4.8)	
5 (Gleason 9+)	3 (15.0)	3 (14.3)	
Pathological T stage, n (%)			0.81 [4]
T2a or less	1 (5.0)	2 (9.5)	
T2b or T2c	12 (60.0)	13 (61.9)	
T3 or more	7 (35.0)	6 (28.6)	
Grade reclassification			**0.05** [5]
Upgrade	6 (30.0)	2 (9.5)	
Same	13 (65.0)	15 (71.4)	
Downgrade	1 (5.0)	4 (19.4)	

[1] One patient did not have a PSA measure at RP. [2] Based on two-sided Student's t-test. [3] Based on an Exact Pearson Chi-Square Test, combining ISUP grade group 4 or more. [4] Based on an Exact Pearson Chi-Square Test. [5] Based on ordinal logistic regression comparing the change over the intervention time between groups. SD: Standard deviation, Q: Quartile, PSA: Prostate-specific antigen, MAG-EPA: monoacylglyceride-conjugated eicosapentaenoic acid; ISUP: International society of urological pathology; RP: radical prostatectomy.
