# Supplementary material for: The gut microbiome-prostate cancer crosstalk is modulated by dietary polyunsaturated long-chain fatty acids
Source: Nat Commun. 2024 Apr 23;15:3431. doi: 10.1038/s41467-024-45332-w (PMC11039720; doi:10.1038/s41467-024-45332-w)
Supplement: Supplementary file 3 — Reporting Summary [file 41467_2024_45332_MOESM3_ESM.pdf]

nature portfolio

Corresponding author(s): Vincent Fradet

Last updated by author(s): Dec 1, 2023

Reporting Summary

Nature Portfolio wishes to improve the reproducibility of the work that we publish. This form provides structure for consistency and transparency in reporting. For further information on Nature Portfolio policies, see our Editorial Policies and the Editorial Policy Checklist.

Statistics

For all statistical analyses, confirm that the following items are present in the figure legend, table legend, main text, or Methods section.

n/a	Confirmed	
☐	☒	The exact sample size (*n*) for each experimental group/condition, given as a discrete number and unit of measurement
☐	☒	A statement on whether measurements were taken from distinct samples or whether the same sample was measured repeatedly
☐	☒	The statistical test(s) used AND whether they are one- or two-sided<br>*Only common tests should be described solely by name; describe more complex techniques in the Methods section.*
☒	☐	A description of all covariates tested
☒	☐	A description of any assumptions or corrections, such as tests of normality and adjustment for multiple comparisons
☐	☒	A full description of the statistical parameters including central tendency (e.g. means) or other basic estimates (e.g. regression coefficient) AND variation (e.g. standard deviation) or associated estimates of uncertainty (e.g. confidence intervals)
☐	☒	For null hypothesis testing, the test statistic (e.g. *F*, *t*, *r*) with confidence intervals, effect sizes, degrees of freedom and *P* value noted<br>*Give P values as exact values whenever suitable.*
☒	☐	For Bayesian analysis, information on the choice of priors and Markov chain Monte Carlo settings
☒	☐	For hierarchical and complex designs, identification of the appropriate level for tests and full reporting of outcomes
☒	☐	Estimates of effect sizes (e.g. Cohen's *d*, Pearson's *r*), indicating how they were calculated

Our web collection on statistics for biologists contains articles on many of the points above.

Software and code

Policy information about availability of computer code

Data collection	no software was used
Data analysis	Statistical Analysis of Metagenomic Profiles (STAMP v2.1.3), Phylogenetic Investigation of Communities by Reconstruction of Unobserved States (PICRUSt, v1.1.4), PAleonto-logical STatitics software (PAST, v3.18).

For manuscripts utilizing custom algorithms or software that are central to the research but not yet described in published literature, software must be made available to editors and reviewers. We strongly encourage code deposition in a community repository (e.g. GitHub). See the Nature Portfolio guidelines for submitting code & software for further information.

Data

Policy information about availability of data

All manuscripts must include a data availability statement. This statement should provide the following information, where applicable:

- Accession codes, unique identifiers, or web links for publicly available datasets
- A description of any restrictions on data availability
- For clinical datasets or third party data, please ensure that the statement adheres to our policy

All tools used for 16S metagenomic are available online; Greengenes [https://mothur.org/wiki/Greengenes-formatted_databases], RDP reference file [https://mothur.s3.us-east-2.amazonaws.com/wiki/trainset9_032012.pds.zip], SILVA reference alignments [https://mothur.org/wiki/Silva_reference_files] and Mothur [https://mothur.org/].

The raw sequence reads generated in this study have been deposited in the NCBI Sequence Read Archive and are directly available under accession code PRJNA1047087 [https://www.ncbi.nlm.nih.gov/sra/PRJNA1047087]. Source data are provided with this paper. Other information is available in supplementary information and otherwise available upon request.

Research involving human participants, their data, or biological material

Policy information about studies with human participants or human data. See also policy information about sex, gender (identity/presentation), and sexual orientation and race, ethnicity and racism.

Reporting on sex and gender	all patients were male (prostate cancer patients)
Reporting on race, ethnicity, or other socially relevant groupings	In the parental clinical trial, patients with prostate cancer were mostly Caucasian male of European ascent (74%) followed by mixed ethnic origin (12%), Caucasian of non-European ascent (7%) and a minor portion of those patients were of African ascent (1%). Race or ethnicity was unknown in 6%.
Population characteristics	Between 55 and 73 years of age. Body mass index between 23 and 32 (kg/m2).
Recruitment	patients were recruited between February 2015 and June 2017
Ethics oversight	This clinical study was approved by our IRB from CHU de Quebec-Universite Laval (2012-1012)

Note that full information on the approval of the study protocol must also be provided in the manuscript.

Field-specific reporting

Please select the one below that is the best fit for your research. If you are not sure, read the appropriate sections before making your selection.

☒ Life sciences ☐ Behavioural & social sciences ☐ Ecological, evolutionary & environmental sciences

For a reference copy of the document with all sections, see nature.com/documents/nr-reporting-summary-flat.pdf

Life sciences study design

All studies must disclose on these points even when the disclosure is negative.

Sample size	For the parental clinical trial, sample size analysis was done using a two-sample t-test for a log-normal geometric mean ratio with a two-sided significance level of 0.05, assuming equal variances, for the primary outcome, the percentage of tumor cells expressing Ki-67. In the study conducted by Aronson et al., (PMID: 22027686) a statistically significant reduction of 32% in the proportion of cells expressing Ki-67 was observed in a group receiving a low-fat diet supplemented with fish oil compared to a control group assigned to a Western diet. We determined that, for the primary outcome, a total of 126 patients (63/group) will provide 90% power to detect a mean ratio of the proportions of cancer cells expressing Ki-67 of ≤0.8, i.e. a 20% difference across groups. Based on previous studies (PMID: 22027686, 19064574), a coefficient of variation of 0.4 was assumed. Considering previous low trial dropout rates (<3%), the target sample size was established at 130 participants (65 per group).
Data exclusions	no data were excluded
Replication	qPCR was used to replicate the 16SrRNA metagenomic data. Mice samples were tested for the relative abundance of several taxa and these target genes were replicated once. For these samples, all attempts at replication were successful. For human fecal samples, qPCR was performed successfully when using a DNA-free Taq polymerase (Boca scientific cat#101005). Replication with FastStart Taq DNA polymerase (Wisent Inc.) yielded inconsistent amplifications for Butyrate kinase and Butyryl CoA transferase amplicons specifically. All other mice experiments represent the full set of independent replicates and the number of biological replicates is described in the figure legends.
Randomization	For animal experiments, prostate tumour cell injections were performed on randomly selected animals. For the parental clinical trial, 130 men were randomized to either MAG-EPA or placebo for seven weeks before radical prostatectomy.
Blinding	For mice experiments, investigators were not blinded since experiments required traceability of the fecal donor sample. For the parental clinical trial, patients, clinicians and study staff were blinded to the intervention; only the pharmacy staff, who was independent of clinical management and outcome adjudication, was unblinded for verification.

Reporting for specific materials, systems and methods

We require information from authors about some types of materials, experimental systems and methods used in many studies. Here, indicate whether each material, system or method listed is relevant to your study. If you are not sure if a list item applies to your research, read the appropriate section before selecting a response.

Materials & experimental systems

n/a	Involved in the study	
x		Antibodies
	x	Eukaryotic cell lines
x		Palaeontology and archaeology
	x	Animals and other organisms
	x	Clinical data
x		Dual use research of concern
x		Plants

Methods

n/a	Involved in the study	
x		ChIP-seq
x		Flow cytometry
x		MRI-based neuroimaging

Eukaryotic cell lines

Policy information about cell lines and Sex and Gender in Research

Cell line source(s)	TRAMP-C2 (ATCC); Pten-/- ; Rbl+/+ and Pten-/-; Rbl-/- prostate cancer cell lines kindly provided by Dr. David Labbe (Department of Anatomy and Cell Biology, McGill University, Montreal, Quebec, Canada)
Authentication	None of the cell lines were authenticated
Mycoplasma contamination	Cells were tested for mycoplasma contamination via qPCR and all cell lined were negative for mycoplasma contamination
Commonly misidentified lines (See ICLAC register)	no commonly misidentified lined were used

Animals and other research organisms

Policy information about studies involving animals; ARRIVE guidelines recommended for reporting animal research, and Sex and Gender in Research

Laboratory animals	6-8 weeks old C57L/6 male mice (The Jackson Laboratories 000664 and Charles rivers 027) were fed ad libitum with chow or low fat diet under a 12h day-night cycle with lights off at 18:00. Relative humidity was maintained between 40% and 60% and temperature was maintained between 20 and 22 Celsius degree.
Wild animals	This study did not involve wild animals
Reporting on sex	Male animals were used to study prostate cancer
Field-collected samples	The study did not involve field-collected samples
Ethics oversight	The RI-MUHC Glen Facility Animal Care Committee and the Institutional Review Board (IRB) of CHU de Quebec - Universite Laval, Canada (#2015112)

Note that full information on the approval of the study protocol must also be provided in the manuscript.

Clinical data

Policy information about clinical studies
All manuscripts should comply with the ICMJEguidelines for publication of clinical research and a completedCONSORT checklist must be included with all submissions.

Clinical trial registration	NCT02333435
Study protocol	The primary study is published here: COMMSMED-23-0186A
Data collection	Patients were recruited between February 2015 and June 2017 followed-up and clinical data was last updated December 2021. Clinical data was collected as part of a regular clinical follow-up of prostate cancer patients at the CHU de Quebec, such as PSA levels, cancer grade, cancer stage and biochemical recurrence. At study baseline (randomization) anthropometry measurements (height, weight) and clinical data such as PSA were collected. The CONSORT checklist of the parental trial is also submitted here.
Outcomes	Prostate cancer proliferation (Ki67 histological staining) as primary outcome. Blood inflammation markers and quality of life (questionnaire), psychosocial functioning (questionnaire) as secondary outcomes. Cancer grade group reclassification (clinical pathology), biochemical recurrence (clinical data) and fatty acid profiles (Gas chromatography) as exploratory outcomes.

Plants

Seed stocks	No seed stocks were used
Novel plant genotypes	No new plant genotypes were produced
Authentication	No plants were used
